# Interaction between physicians and the pharmaceutical industry: A scoping review for developing a policy brief

**DOI:** 10.3389/fpubh.2022.1072708

**Published:** 2023-01-12

**Authors:** Ehsan Zarei, Amir Ghaffari, Ali Nikoobar, Shayan Bastami, Hasan Hamdghaddari

**Affiliations:** ^1^Department of Health Service Management, Virtual School of Medical Education and Management, Shahid Beheshti University of Medical Sciences, Tehran, Iran; ^2^School of Medicine, Shahid Beheshti University of Medical Sciences, Tehran, Iran; ^3^Social Determinants of Health Research Center, Shahid Beheshti University of Medical Sciences, Tehran, Iran

**Keywords:** conflict of interest, medical ethics, pharmaceutical industry, transparency, gift giving, physician-industry interaction

## Abstract

**Background:**

Payments to physicians by the pharmaceutical industry are common, but recent evidence shows that these payments influence physician prescribing behavior in the form of increased prescription of brand-name drugs, expensive and low-cost drugs, increased prescription of payer company drugs, etc. Considering that these payments increase drug costs for patients and health systems, there is a public interest in controlling them. Therefore, this study aimed to identify and propose policy options for managing physician-pharmaceutical industry interactions in the context of Iran's health system.

**Methods:**

In the first phase, a systematic search was conducted to identify relevant policies and interventions in Web of Science, PubMed, and ProQuest databases from 2000 to 2022. Then, the opinions of the research team and an expert group (physicians, health policy and transparency experts, and industry representatives) were used to categorize the interventions and propose policy options along with their advantages, disadvantages, and implementation considerations.

**Results:**

In the search, 579 articles were retrieved, and 44 articles were found suitable for the final analysis. Twenty-nine interventions and strategies were identified, and based on these; Five policy options were identified: prohibition, restriction, physician self-regulation, voluntary industry disclosure, and mandatory industry disclosure.

**Conclusion:**

The proposed policies in our study include advantages, challenges, and implementation considerations based on up-to-date evidence that can help policymakers use them to manage COI in physician-pharmaceutical industry interactions in Iran's health system. A combination of measures seems to help manage COI: firstly, using self-regulating physicians and industry to institutionalize transparency, and in the next step, implementing mandatory industry disclosure policies and establishing restrictions on some financial interactions.

## Introduction

The pharmaceutical industry is a strategic partner in advancing the goals of the healthcare sector (1). Physicians attend professional meetings with pharmaceutical representatives, participate in research, and participate in development and investment for health-related industries, all of which are often important opportunities for advancing medical knowledge and patient care (2). Physicians and the industry have a common interest in advancing medical knowledge. Nevertheless, the physician's primary goal is to promote their patient's interests, while the industry's goal is to promote profitability and uses all available tools to promote its products, which are not necessarily in line with the patient benefit (1, 2).

Therefore, these interactions can create conflicts of interest (COI) that affect clinical judgments, prescribing, research, education, and treatment outcomes. There is considerable evidence that this COI often favors companies (3). Some studies show considerable concern about these interactions, especially in financial interest cases, as such links potentially lead to systematic biases in patient care (4). Physicians are the gatekeepers determining how money is spent in the health system. Hence, they are the target group of marketing activities of the pharmaceutical industry (5), and potential COI, real or perceived, is pervasive in this area (2).

Payment by the pharmaceutical industry to physicians is the most common form of physician-industry relationship, which is in the form of cash (for consulting services, lectures, travel, accommodation, etc.) or non-cash such as meals, gifts, stocks, licenses, etc. (6, 7). About 90% of pharmaceutical companies advertising costs are allocated to physicians and other prescribers (3). Industry payments to physicians and teaching hospitals in the United States, excluding scientific research funding, amounted to $3.6 billion in 2019 (8). Almost half of the physicians receive annual payments from the industry (9). The findings of a systematic review showed that financial relationships between physicians and the pharmaceutical industry are common in low- and middle-income countries (10).

There is not much documentary evidence about these payments in Iran. Tabrizi et al. claimed that in Iran, pharmaceutical companies choose their target physicians among health policymakers who play an essential role in pharmaceutical decisions to ensure the success of their marketing (11). According to a news report in 2019, there are cash incentives, free drug samples, and various rewards to doctors, such as “foreign trips” for prescribing products of a particular company in Iran's health system (12). The financial relationship of physicians with pharmaceutical companies has also been mentioned as one of the important reasons for the irrational prescription of drugs in Iran (13, 14). In Ebrahimi et al.'s study, 90% of breast cancer specialists stated that they participated in congresses sponsored by pharmaceutical companies to introduce new drugs (15).

Payments by the pharmaceutical industry to physicians have some negative consequences. These payments can affect the independent clinical decision-making of physicians and thus endanger the quality of patient care, increase healthcare costs and reduce patients' trust in physicians and the health system (1, 6, 16, 17). Almost all published studies reported a positive relationship between payments and changes in physician prescribing behavior (1, 6, 7, 18).

It has been reported as the consequences of the industry's payments to physicians, such as increased use of brand-name drugs, increased prescription of medicines produced by the paying company, prescription of low-value and expensive drugs, preference and rapid prescription of new drugs, requests to add promoted drugs to the country's official list of drugs and finally increasing drug costs (6, 7, 16, 19). A study in the United States showed that a 10% increase in pharmaceutical industry payments to physicians is associated with a 1.3% increase in medical costs and a 1.8% increase in drug costs (20).

But physician-industry interactions also have defenders. They argue that industry funding leads to the development of drugs and devices that benefit patients. Product development, production, and marketing depend on the industry and are not possible only with government funds. The industry also needs physicians to test products to use those products in clinical trials. Humanity owes a huge collection of life-saving drugs and surgical devices to the cooperation of industry and physicians (21). In general, physician-industry relationships are not inherently good or bad. There are positive interactions between physicians and the industry that help medical advances. Physicians are and should be compensated for this work, but such interactions may create the potential for bias (22). Fear of industry bias should not prevent beneficial clinical interactions or innovative research that helps patients. on the other hand, maintaining public trust in the health system is also important (23).

That is why these interactions must be properly structured and managed. In addition, some inappropriate interactions and payments need to be monitored and addressed (22). With the lack of effective monitoring and management of COI, such interactions may eventually threaten the integrity, justice, and sustainability of health systems and negatively affect patients (1). Therefore, a middle ground should be sought to control physician-industry interactions; Neither a complete prohibition can help nor a complete freedom without transparency of these interactions (22). A more balanced and transparent interaction between physicians and the pharmaceutical industry approach would recognize beneficial collaborations while eliminating inappropriate relationships such as sham payments, promotional activities, meals provided by industry representatives, and speakers' bureau activities.

Therefore, there is a public interest in controlling them (7), and policymakers worldwide are looking for effective strategies to protect physicians from the industry's undue influence and manage COI from unregulated and non-transparent interactions (5, 24). To our knowledge, a comprehensive guide on proposed interventions and policies regarding physician-industry interactions was not found. Therefore, this study aimed to identify and propose policy options for managing physician-pharmaceutical industry interactions in the context of Iran's health system.

## Methods

### Eligibility criteria

The following criteria were used to select studies: (1) articles that dealt with specific interventions, strategies, or policies regarding managing financial interactions between physicians and pharmaceutical companies; (2) All types of articles such as reviews, originals, etc., except Conference articles and book chapters; (3) English language; (4) Studies whose target or discussed population for intervention or strategy proposal were physicians; (5) articles whose full text was available.

### Information sources

A literature search was conducted on August 30, 2022. Three databases, PubMed, ProQuest, and Web of Science, were searched. The time range was considered from January 1, 2000, to 30, August 2022.

### Search

The search was conducted using the following keywords: doctor, physician, pharmaceutical, industry, interaction, relation, collaboration, payment, influence, and conflict of interest (see the complete search strategy in Appendix 1).

### Selection of sources of evidence

We used the PRISMA model for screening and selecting articles (25). All retrieved records were exported to EndNote X9. After removing duplicate articles, the titles and abstracts of the remaining articles were reviewed by two team members (EZ and AG) independently. After determining the relevance, the full text of the articles was retrieved for detailed review and data extraction.

### Data charting process

Each article was read by two authors independently, and relevant strategies/interventions were extracted and then discussed and agreed to be mentioned in the data form.

### Data items

Only strategies/interventions proposed in studies or tested in real-world settings were extracted in this review.

### Synthesis of results

Data extraction and synthesis were done in two stages. First, the authors extracted interventions and strategies from the articles. These were then categorized into proposed policy options. There are two approaches to managing COI: eliminating all situations of COI by prohibitions and restrictions and controlling COI by transparency and disclosure of interactions (26). We first categorized the proposed interventions and strategies based on these approaches: 1- prohibition and restriction and 2- disclosure. In the next step, we realized that disclosure could be done from two sides: the payer (pharmaceutical companies or the industry) and the receiver (physicians). Also, industry disclosure is now voluntary and mandatory so that these options can be separated according to their specific implementation considerations.

In the second stage, we formed a team of experts. These people were selected purposefully and related to the research subject, including two physicians, two industry representatives, and three health policy experts (two of them are researchers in the field of transparency). We shared our proposed policy options with the experts' team, who confirmed our classification. In the next step, we asked the experts to tell us their opinions on these five options: “What are your opinions about the advantages and disadvantages of this policy option? What considerations should be taken into account in implementing this policy?” The interview was conducted online, and the audio was recorded. Some of the interviewees said were in the evidence, and some new points were added, especially considering the context of Iran's health system.

## Results

### Selection of sources of evidence

**Figure 1:**
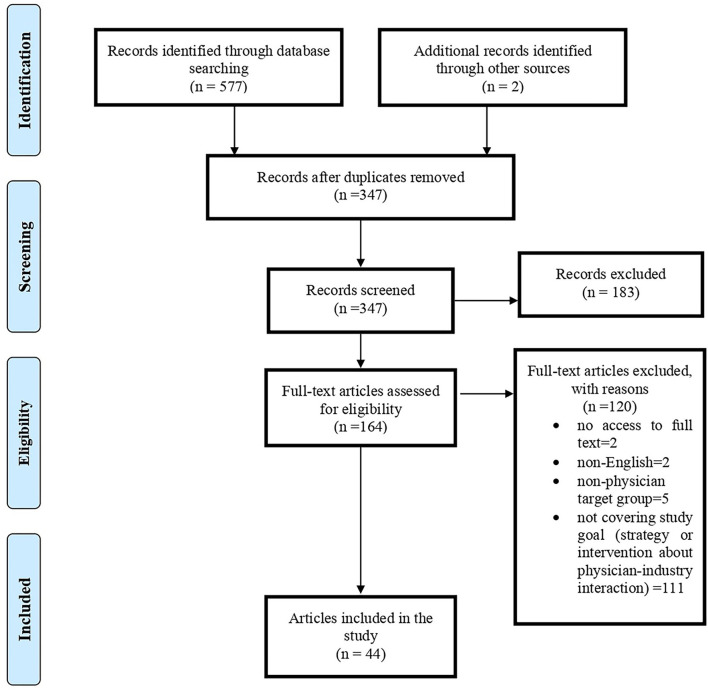
PRISMA flowchart of systematic literature review.

In the search, 579 articles were retrieved; after removing duplicate titles (232 articles), the titles and abstracts of the remaining articles were reviewed. From the remaining 164 articles, 44 were found suitable for the research topic (Figure 1). Of 44 papers, 32% (*N* = 14) was original, 57% (*N* = 25) was review articles (including seven systematic reviews), four editorial, and one RCT. Most articles were published in 2017 and 2021 (*N* = 6); generally, 70% of included articles were published in recent 10 years. Regarding affiliation of authors, 48% (*N* = 21) were related to the USA and then to Lebanon (*N* = 4) and Germany (*N* = 3). In addition, we had articles from 15 countries, including one from Iran. BMJ Open (*N* = 5), JAMA (*N* = 4), PLoS ONE (*N* = 3), Health Policy (*N* = 3), and Annals of Internal Medicine (*N* = 3) provided the most paper for our study. The characteristics of included papers are presented in Table 1.

**Table 1:** Characteristics of included articles.

**References**	**Country**	**Article type**	**Publication source**
Stoll et al. (4)	Germany	Original	BMJ Open
Mitchell et al. (7)	USA	Review	Journal of health politics, policy, and law
Ozieranski et al. (27)	UK	Review	BMJ Open
Rose et al. (28)	USA	RCT	Organizational Behavior and Human Decision Processes
Ansari (17)	USA	Original	Social Science and Medicine
Moriarty et al. (29)	Ireland	Original	Health Policy
Mulinari et al. (30)	Sweden	Original	Health Policy
Brown (31)	USA	Editorial	American Family Physician
Kang (23)	USA	Review	Rheumatic Disease Clinics
Kanter et al. (32)	USA	Original	JAMA
Garattini and Padula (33)	Italy	Editorial	European Journal of Health Economics
Martínez (34)	Colombia	Editorial	Revista Colombiana de Cardiología
Grundy et al. (16)	Australia	Review	Health Policy
Fadlallah et al. (10)	Lebanon	Systematic Review	European Journal of Public Health
Zezza and Bachhuber (35)	USA	Original	PLoS ONE
Mulinari et al. (36)	Sweden	Original	BMJ Open
Fickweiler et al. (18)	Netherlands	Systematic Review	BMJ Open
King and Bearman (24)	USA	Original	Social Science and Medicine
Makowska (37)	Poland	Original	PLoS ONE
Brax et al. (38)	Lebanon	Systematic Review	PLoS ONE
Larkin et al. (39)	USA	Original	JAMA
Nissanholtz-Gannot and Yankellevich (40)	Israel	Original	Israel Journal of Health Policy Research
Keller et al. (41)	Germany	Review	Croatian Medical Journal
Fadlallah et al. (19)	Lebanon	Systematic Review	PLoS ONE
Gupta et al. (42)	India	Original	Journal of Pharmacy and Bioallied Sciences
Breault et al. (43)	USA	Review	Hospital Medicine Clinics
Kirschner et al. (3)	USA	Review	Annals of Internal Medicine
Alkhaled et al. (44)	Lebanon	Systematic Review	BMJ Open
Hwong et al. (45)	USA	Systematic Review	Journal of Law, Medicine and Ethics
Sahm (5)	Germany	Review	Medicine Health Care and Philosophy
Sillup et al. (11)	Iran	Review	International Journal of Healthcare Management
Albersheim and Golan (46)	Canada	Review	Israel Medical Association Journal
Immelt et al. (47)	USA	Review	Annals of Thoracic Surgery
Nakayama (21)	USA	Editorial	American Surgeon
Grande (48)	USA	Review	Journal of General Internal Medicine
Fugh-Berman et al. (49)	USA	Original	Journal of Continuing Education in the Health Professions
Ross et al. (50)	USA	Original	JAMA
Thomas (51)	USA	Review	The Virtual Mentor
Studdert et al. (52)	USA	Review	New England Journal of Medicine
Wager (53)	UK	Review	BMJ
Coyle (2)	USA	Review	Annals of Internal Medicine
Coyle (56)	USA	Review	Annals of Internal Medicine
Wazana and Primeau (54)	Australia	Review	Medical Journal of Australia
Wazana (55)	Canada	Systematic Review	JAMA

### Results of individual sources of evidence

A list of elicited strategies and interventions for managing COI arising from the physicians-pharmaceutical industry interaction is presented in Table 2.

**Table 2:** Strategies and interventions extracted from the systematic review for managing COI of the physician-pharmaceutical industry.

	**Strategies and interventions**	**References**
**Strategies**		
1	Prohibition of all financial interactions	(2, 7, 16, 29, 33, 41)
2	Prohibition of financial interactions affecting prescription	(48)
3	Prohibiting the presence of physicians with financial COI in pharmaceutical decision-making committees	(21, 43, 46)
4	Distinguishing between administrative and personal gifts	(19, 53)
5	Prohibition of non-service gifts	(54)
6	Acceptance of some gifts that are for the benefit of the patient	(2, 8, 48, 53)
7	Organizing pharmaceutical representatives and developing a code of ethics for them	(48)
8	Developing guidelines for the physicians-pharmaceutical representatives' interaction	(55)
9	Restrictive government regulations	(5, 19)
10	Establishing a COI committee at the hospital level	(43)
11	Physician-industry financial interaction through a hospital or academic center	(47)
12	Strengthening regulatory structures and independence from the industry	(16)
13	Voluntary codes of conduct of pharmaceutical companies	(53)
14	Transparency and openness of physician-pharmaceutical industry interactions	(54)
15	Physicians' self-regulation	(7, 8, 24, 51)
16	National disclosure system of physician-industry relations	(10)
17	Compilation and strengthening of codes, guidelines, and ethical guidelines for physician-industry interactions	(2, 5, 37, 42, 48)
18	Clear and transparent procedures regarding accepting and disclosing gifts, sponsoring travel, and continuing education	(54)
**Interventions**		
1	Prohibition of gifts, payment for lectures, travel, and direct financial support of continuing education programs	(7, 8, 16, 21, 24, 30, 48)
2	Prohibition of cash payments above a certain threshold	(5, 17, 33, 53)
3	Prohibition of receiving drug samples	(21, 31, 38, 44)
4	Prohibition of receiving promotional materials	(10, 38, 44)
5	Prohibition of meeting with pharmaceutical representatives in clinical settings	(16, 31, 44, 46)
6	Limiting interactions between physicians and pharmaceutical representatives	(10, 18, 24, 38, 39)
7	Educational programs on the legal and ethical aspects of physician-pharmaceutical industry interactions	(10, 18, 19, 23, 34, 38, 44, 46, 49, 56)
8	Disclosure letter/ oral disclosure of interactions during admission or patient consultation	(19, 28, 46)
9	Disclosure of gifts and payments by physicians	(3, 24, 33, 37, 46)
10	Mandatory public disclosure of the industry	(3, 7, 16, 24, 45)
11	Public disclosure through voluntary industry self-regulation	(11, 16, 27, 29, 30, 36)

### Synthesis of results

**Figure 2:**
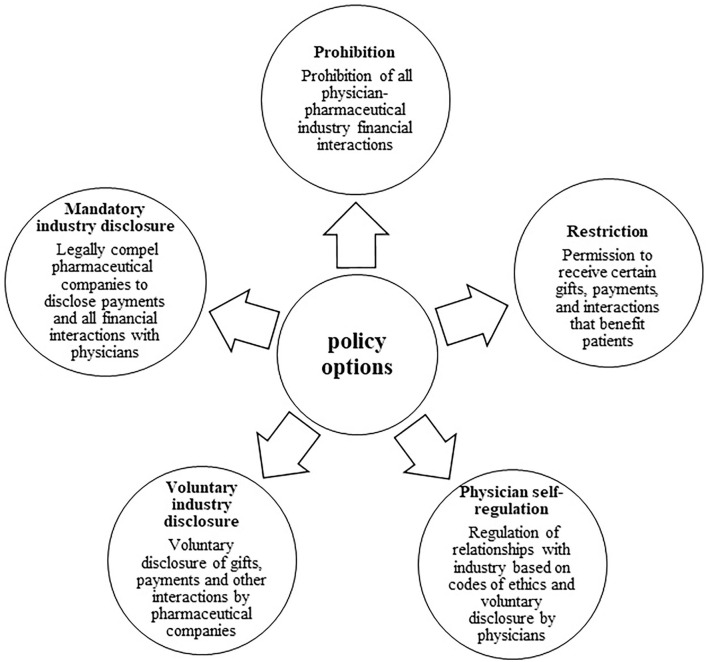
Policy options for managing the COI arising from the physician-industry interaction.

In the second phase, with the collaboration of the experts' team, five policy options were proposed (Figure 2):

- Prohibition: ban on all physician-pharmaceutical industry financial interactions.
- Restriction: permission to receive certain gifts, payments, and interactions that benefit patients.
- Physician self-regulation: regulation of relationships with industry based on codes of ethics and voluntary disclosure by physicians.
- Voluntary industry disclosure: disclosure of gifts, payments, and other interactions by pharmaceutical companies on an optional and voluntary basis.
- Mandatory industry disclosure: all pharmaceutical companies must disclose payments to physicians by law.

The policy options' advantages, disadvantages, and implementation considerations are presented in Tables 3–7.

**Table 3:** Advantages, disadvantages, and implementation considerations of policy options: 1. Prohibition.

**Advantages**	**Disadvantages**	**Implementation considerations**
• Preventive approach to unethical behavior (7, 23) • Protection of physicians' professional judgment and patient care (2) • Easier justification of this action in line with the public interest (7) • Attracting public trust in the health system • Management of pharmaceutical costs • The threat caused by regulations can be the motivation to change professional behavior (48)	• Loss of industry financial support for educational and research programs, especially continuing education programs (29) • Political impossibility (unpopular among affected groups such as physicians) (7) • The information gap is caused by the lack of communication between physicians and pharmaceutical representatives (38) • The need for strict supervision and clear legal sanctions (41) • Limited effect (40) • Payment by unobservable methods (such as cryptocurrency)	• Requires an official law approved by the parliament • Lobbying and negotiating with various influential institutions and groups for the approval of the law • Compilation of executive and penal regulations for non-compliance with the prohibition law (7, 48) • Close monitoring of law enforcement and support for whistleblowers to report violations (52) • Launching an up-to-date pharmaceutical information system to compensate for the lack of relationship between physicians and pharmaceutical representatives

**Table 4:** Advantages, disadvantages, and implementation considerations of policy options: 2. Restriction.

**Advantages**	**Disadvantages**	**Implementation considerations**
• Easier justification for accepting gifts that enhance medical performance or knowledge and are of moderate value to the public (2) • Helping poor patients through the distribution of free drug samples (2, 8) • Updating doctors and familiarizing them with new technologies and drugs through interaction with pharmaceutical representatives (19) • A helpful policy for the short term and before the implementation of the prohibition	• Lack of consensus on the appropriateness of gifts and different valuations of a product or service in different environments and societies (2) • After the free medicine runs out, the physician's prescription is usually of the same brand, which leads to an increase in costs (2, 8) • Misappropriation of information prepared by the industry (2, 8). • Requires strict monitoring of the implementation of restrictions	• Setting a threshold above which the payment should be declared a COI (33). • Drafting transparent and clear policies regarding the acceptance of gifts and payments • Establish COI committees at the ministry and medical universities level to formulate exemptions and monitor their implementation according to local conditions.

**Table 5:** Advantages, disadvantages, and implementation considerations of policy options: 3. Physician self-regulation.

**Advantages**	**Disadvantages**	**Implementation considerations**
• Accuracy in making decisions about interaction with the industry (7) • Obtaining financial support from the industry in education and research and assisting in patient care • Improving the patient's trust in the doctor and stronger doctor-patient relationships (3) • Less bias in clinical decisions (28) • Cultivation for the institutionalization of transparency and disclosure of COI in the health system	• Less likely to succeed because relationships with industry are always attractive to physicians (7) • Doubts about the validity, transparency, comprehensibility, and completeness of the disclosed data (3) • Moral license for all financial interactions (24, 48) • Teaching ethics and relying on ethical codes alone is not enough to prevent violations and biases (23)	• Compilation of ethical codes for physician-industry interactions by the Medical Council or the Ministry of Health • Teaching medical students how to manage COI and how to interact with the industry • Training of COI management and how to interact with the industry to members of medical professional associations in annual meetings (56) • Determining minimum standards for gifts and payments that must be disclosed • Creating a system to record voluntary disclosure cases • The reward for compliance with ethics and professional norms (48)

**Table 6:** Advantages, disadvantages, and implementation considerations of policy options: 4. Voluntary industry disclosure.

**Advantages**	**Disadvantages**	**Implementation considerations**
• A step toward institutionalizing transparency in the health system (29) • The optional disclosure of physicians' names increases the desire to disclose data • It derives its legitimacy from the relations between the stakeholders, not from imposition by the government (40) • Attracting public trust and gaining credibility for the industry	• Limited access to payment data and their scatteredness on companies' websites (30) • Different reporting methods between companies (overall and without payment details, in PDF format, unanalyzable) (16, 27, 30) • Anonymity of recipients (29) • The quality and validity of disclosed data, the incompleteness of payment data, and their complete non-disclosure (for example, food and beverages) (27, 30, 36) • In the absence of robust enforcement mechanisms, such as sanctions, it has little effect (40) • Sometimes used to prevent the application of government regulations (40) • Unfair competition; Companies that disclose payment data may be at a competitive disadvantage • The responsibility of transparency is transferred from the health system to the industry	• Drafting regulations and minimum disclosure standards for pharmaceutical companies • Compliance of disclosure guidelines with personal data protection laws in the country • The need to create a culture for the willingness of physicians and companies to disclose data with the recipient's name • Rewards and incentives for leading companies in payment transparency and disclosure • Using the capacity of civil activists and non-governmental organizations to pressure and encourage the industry to disclose

**Table 7:** Advantages, disadvantages, and implementation considerations of policy options: 5. Mandatory industry disclosure.

**Advantages**	**Disadvantages**	**Implementation considerations**
• Influence on the physicians' behavior to reduce financial interactions with the industry (4) • Absence of feeling of injustice among physicians regarding information disclosure (4) • Public access to information, easier comparison, and interpretation, reducing the risk of unfair damage to reputation (4) • Helping patients get better information about physicians, choosing a physician, and making treatment decisions (45) • Increasing transparency and improving public trust in the health system • Helping to better monitor physicians by following up on data disclosed by the media (48) • Deterrent effect for suspicious and unethical payments (32)	• Difficulty in interpreting disclosed information for consumers (patients) (16, 48) • Its political aspect is bold (16) • Disclosure does not determine whether physician-industry interactions are appropriate • Decrease in patients' trust (32)	• It is necessary to raise the issue in general regulations (law by the parliament) • Establishment of the cooperation office of the Ministry of Health and Industry to implement the policy • Creating a searchable and user-friendly open payment system available to the public (45) • Determining the types and nature of payments and financial exchanges, determining the items that do not need to be disclosed (standardization of inputs and data) (50) • Determination of crimes and sanctions for non-disclosure and incomplete reporting (16) • Education to help patients understand data and use it to make healthcare decisions (35)

## Discussion

This study was conducted to identify policy options for managing physician-industry interactions in Iran, and five policy options were proposed. In this section, we have discussed these options with an emphasis on their implementation considerations.

### Prohibition

There is a strong relationship between receiving industry benefits and supporting their products (2). Since the primary goal of pharmaceutical representatives is to promote their products—not to serve the interests of patients—so the best tactic for physicians is to avoid them (33). A concern often raised in defense of these interactions is that meetings between pharmaceutical representatives and physicians accompany the presentation of information about new drug products and allow busy physicians to stay up-to-date more efficiently (7). If the prohibition policy is implemented, the relationship between physicians and pharmaceutical representatives will be cut off, which should be addressed by designing and setting up an updated drug information system. Prohibition policy has a preventive approach toward unethical behavior and possible adverse consequences of physician-industry interaction (7). Passing and implementing the law prohibiting physician-industry interaction has many implementation challenges. Prohibition of such interactions requires legislators to formulate and pass clear and precise laws to ban all types of industry payments to physicians (7), which will probably face reactions from the medical community. Monitoring the implementation of the prohibition law is challenging and may transfer these payments to unobservable ways (such as cryptocurrency). Government regulators can reduce physician-industry COI by increasing sanctions for such activities.

### Restriction

An alternative policy to the prohibition is to restrict physician-industry interactions and distinguish between acceptable and unacceptable payments and gifts. Evidence shows that restrictive policies may have a positive effect on improving the prescribing behavior of physicians (38). In implementing this policy, receiving inexpensive gifts for use in the physician's office (such as notebooks or pens) and items related to patient care (drug samples and medical booklets) that do not hurt care is not prohibited, but gifts and payments such as recreational events for physicians, payment for lectures or free meals are strictly prohibited due to the increased potential for COI (2, 7). The Iranian Medical Council has also declared it acceptable gifts that benefit patients (such as drug samples for poor patients) (57). Also, according to Iran's Pharmaceutical Code of Ethics, it is allowed to accept low-value gifts, such as calendars, pens, etc., from pharmaceutical companies (58).

Implementing restrictive policies requires that medical universities and hospitals have clear and transparent procedures and guidelines for accepting gifts and payments that specify what is acceptable and prohibited. Necessary executive action determines a “threshold” above which payment should be declared a financial COI. This difficult task depends on the countries' culture, history, and wealth (33). It is suggested that COI committees be formed at the health ministry and medical sciences university level to formulate exemptions and monitor their implementation according to local conditions and the extent of the relationship with the industry.

### Physicians' self-regulation

The medical profession traditionally relies on self-regulation to implement the ethical standards of medicine and protect the patient's interests (51). When the primary goals of patient care, education, and clinical research may be threatened by financial or other secondary interests, physicians have a responsibility to self-regulate; as such, COI may undermine public trust in the physicians (8). Physicians often believe that a conscious commitment to ethical behavior and professionalism protects them from undue industry influence (59). Therefore, physicians' self-regulation relies on strengthening moral norms (60). Self-regulatory tools include voluntary disclosure, ethical guidelines, and educational interventions to reduce COI. Physician self-regulation can be implemented as a transitional policy to institutionalize a transparency culture before enforcing mandatory transparency laws.

In the Pharmaceutical Code of Ethics, as well as the Professional Ethics Guide of the Iranian Medical Council, there are cases of how to interact with pharmaceutical companies. For example, it is prohibited to give cash and non-cash gifts to physicians by pharmaceutical companies and to accept any money for travel and accommodation costs for vacations, participation in conferences, seminars, workshops, and continuing education programs from companies and industries, for themselves or their families (57). To implement this policy, it is necessary to compile a complete ethical guide by the Iranian Medical Council or the Ministry of Health to determine acceptable cases and ethical behaviors in physician-industry interactions.

Disclosure of physician-industry interactions can be made voluntarily on the physician's website or a central website. Another approach for physicians is to disclose their interactions with the industry through printed materials or a disclosure form during admission, an informed consent form, or verbally during consultation and examination of patients (19, 28). Physician disclosure does not lead to a behavior becoming ethical, but it is a step toward promoting ethical behavior (3); It can also lead to the promotion of the view among physicians that whatever is disclosed is no longer a problem (a moral license) (24). To promote a transparency culture, incentives are also determined to comply with ethics and professional norms (48).

One of the most critical executive measures in the physician self-regulation policy is empowerment, informing, and training about interactions with the industry and its consequences for physicians. Training is available at two academic levels for medical students and continuing education programs for practicing physicians. Educating members of medical professional societies on issues related to physician-industry interactions can be accomplished at annual meetings (56). These programs aim to help physicians better understand the COI associated with accepting gifts and other financial incentives and their potential impact on patient care (48). Evidence shows that educational programs are effective in increasing awareness and changing the attitude of physicians regarding their sensitivity toward interaction with pharmaceutical companies (18, 49).

### Voluntary industry disclosure (self-regulation)

Disclosure of payments and financial interactions with pharmaceutical companies can be voluntary (self-regulation) by the industry. Self-regulation allows the industry to design, implement, and monitor payment disclosure rules (27). In this policy, pharmaceutical companies are required to disclose their payments to physicians annually (30), and a trade body (such as the Pharmaceutical Manufacturers Association) supervises the implementation of the policy (16). The European Federation of Pharmaceutical Industries and Associations (EFPIA) requires pharmaceutical companies to report physician payments (36). In Australia, the Pharmaceutical Industry Association requires member companies to publicly report payments to physicians and medical facilities (16). The industry's voluntary disclosure policy is implemented in more than 30 European countries (27, 30).

Implementing this policy requires formulating regulations of minimum disclosure standards for pharmaceutical companies, which the health system can determine. The data can be published on a central platform (for example, in the UK on the Disclosure UK database) or on the company's website. Companies have considerable discretion over publishing and accessing data, leading to different reporting or general reports without detailing payments (30). The incompleteness of payment data and lack of full disclosure of them (for example, meals) is a significant flaw of the industry's self-regulation mode (27, 30). The findings of a study showed that in 23 European countries with a self-regulation approach, disclosures are published in PDF documents on companies' websites, preventing the public from understanding payment patterns (27).

One of the critical shortcomings of industry self-regulation is that it makes disclosure by paying companies conditional on recipients' consent, and a physician can request anonymity (27, 36). In this regard, there is a need to adapt the data disclosure criteria to the personal information protection law in the country. Efforts should be made to create a culture of the willingness of physicians and companies to disclose data with names and details. In this regard, individuals and companies can use financial and non-financial incentives. Due to the voluntary nature of disclosure and the less willingness of physicians and industry to disclose information, the capacity of non-governmental organizations and civil activists can be used to pressure and encourage disclosure.

### Mandatory industry disclosure

In implementing the policy of mandatory disclosure of payments by the industry, a government institution such as the Ministry of Health is responsible for implementation and monitoring (16). The most famous policy in this field is the Sunshine Payment Act, which was passed in 2010 in the United States, and a system called “open payment” was launched to implement this law in 2013, which requires pharmaceutical companies to disclose their payments to physicians in this system (3). The French initiative is the Bertrand law and the Transparency Santé system (16). Mandatory disclosure regulations have also been implemented in countries such as Japan, Turkey, Portugal, Greece, Denmark, Romania, Latvia, and Slovenia (16).

Mandatory industry disclosure must be proposed in the public regulations, and the law must be approved in the parliament, which may be influenced by the lobby of the physician's union and groups. Also, disclosure standards and criteria, along with relevant guidelines, should be developed, and a searchable and user-friendly open payment system with public access should be designed (45). In this regard, there is a need for continuous cooperation and interaction between the Ministry of Health and industry representatives, and a joint office should be established for this purpose. One of the important measures of this office is to determine the types and nature of payments and financial exchanges, to select the items that do not need to be disclosed, and in general, to standardize inputs, data, time frame, and publishing methods (50).

Types of reportable payments include cash, gifts, and stock in the form of consulting fees, food, and beverage, payments for participation in continuing education programs and lectures, grants, and research payments made directly or indirectly (through a third party) to physicians. Also, ownership interests, such as shares in pharmaceutical companies, must be reported. Financial interactions exempt from disclosure can include drug samples and items with a value of <$10 per transaction or $100 per year (3). Physicians preview the data before it is publicly released and, if necessary, object to corrections.

Supporters of public reporting policies support it as a tool to manage industry influence and COI and believe that public reporting acts as a deterrent to inappropriate relationships between physicians and industry (16). Evidence shows that public disclosure reduces the recipients of financial benefits from companies (3).

## Limitations

This systematic review had limitations. At the time of the search, we, unfortunately, did not have access to the Scopus database. Therefore, we may have missed some relevant studies. We only included English-language studies, so strategies and interventions published in non-English-language articles may have been unique that we did not consider.

## Conclusion

The proposed policies in our study include advantages, challenges, and implementation considerations based on up-to-date evidence that can help policymakers to manage COI in physician-pharmaceutical industry interactions in Iran's health system. Transparency is an essential part of resolving COI. Disclosure of industry payments to physicians is necessary but insufficient for addressing COI and ensuring the independence of physicians, regulators, and health systems. Disclosure of COI does not necessarily lead to eliminating or preventing bias in clinical decision-making. Paradoxically, transparency may normalize financial COI or increase their influence through a moral license. Therefore, transparency should be accompanied by policies that seek to reduce or eliminate some of these COIs. Although the government must play an essential role through regulation and supervision, physicians must rely on self-regulation and professional ethics to rid the profession of undue commercial influence. It seems that a combination of measures can help to reduce the adverse effects of COI: firstly, using self-regulation of physicians and industry to institutionalize transparency, and in the next step, implementing mandatory industry disclosure policies and establishing restrictions on some financial interactions.

## Data availability statement

The original contributions presented in the study are included in the article/supplementary material, further inquiries can be directed to the corresponding author.

## Author contributions

EZ and AG conceived and designed the study. EZ, AG, AN, and SB conducted the literature searches and extracted the data. HH performed the interviews. EZ and AN wrote the manuscript. HH and SB revised the manuscript. All authors contributed to the article and approved the submitted version.

## Appendix 1

PubMed

#1 (doctor*[Title]) OR (physician*[Title])

#2 ((drug*[Title]) OR (pharmaceutical [Title])) OR (industry [Title])

#3 (((((((((interact*[Title/Abstract]) OR (relation*[Title/Abstract])) OR (link*[Title/Abstract])) OR (collaboration*[Title/Abstract])) OR (contact [Title/Abstract])) OR (influence*[Title/Abstract])) OR (payment [Title/Abstract])) OR (tie [Title/Abstract])) OR (conflict of interest [Title/Abstract]))

#4 ((((((((control [Title/Abstract]) OR (polic*[Title/Abstract])) OR (strateg*[Title/Abstract])) OR (intervention*[Title/Abstract])) OR (solution*[Title/Abstract])) OR (law [Title/Abstract])) OR (regulat*[Title/Abstract])) OR (recommendation*[Title/Abstract])) OR (legislat*[Title/Abstract]) (((((drug*[Title]) OR (pharmaceutical [Title])) OR (industry [Title])) AND ((doctor*[Title]) OR (physician*[Title]))) AND ((((((((((interact*[Title/Abstract]) OR (relation*[Title/Abstract])) OR (link*[Title/Abstract])) OR (collaboration*[Title/Abstract])) OR (contact [Title/Abstract])) OR (influence*[Title/Abstract])) OR (payment [Title/Abstract])) OR (tie [Title/Abstract])) OR (conflict of interest [Title/Abstract])))) AND (((((((((control [Title/Abstract]) OR (polic*[Title/Abstract])) OR (strateg*[Title/Abstract])) OR (intervention*[Title/Abstract])) OR (solution*[Title/Abstract])) OR (law [Title/Abstract])) OR (regulat*[Title/Abstract])) OR (recommendation*[Title/Abstract])) OR (legislat*[Title/Abstract])) Filters: English, from 2000 – 2022

Filters: English, from 2000 – 2022

--------------------------------------------------------------------------------------------------------------------

WOS

#1 (TI=(doctor*)) OR TI=(physician* )

#2 ((TI=(drug*)) OR TI=(pharmaceutical)) OR TI=(industry)

#3 ((((((((TS=(interact*)) OR TS=(relation* )) OR TS=(link*)) OR TS=(collaboration*)) OR TS=(contact )) OR TS=(influence*)) OR TS=(payment )) OR TS=(tie )) OR TS=(conflict of interest)

#4 ((((((((TS=(control )) OR TS=(polic*)) OR TS=(strateg*)) OR TS=(intervention*)) OR TS=(solution*)) OR TS=(law )) OR TS=(regulat*)) OR TS=(recommendation*)) OR TS=(legislat*)

#5: #1 AND #2 AND #3 AND #4

2000–2022

------------------------------------------------------------------------------------------------------------------

ProQuest:35

((ti(doctor*) OR ti(physician*)) AND PEER(yes)) AND ((ti(industry) OR ti(drug*) OR ti(pharmaceutical)) AND PEER(yes)) AND ((ab(interact*) OR ab(relation*) OR ab(link*) OR ab(collaboration*) OR ab(contact) OR ab(influence*) OR ab(payment) OR ab(tie) OR ab(conflict of interest)) AND PEER(yes)) AND ((ab(control) OR ab(polic*) OR ab(strateg*) OR ab(intervention*) OR ab(solution*) OR ab(law) OR ab(regulat*) OR ab(recommendation*) OR ab(legislat*)) AND PEER(yes))

2000–2022

-------------------------------------------------------------------------------------------------------------------
